# Vitamin D levels and risk of ocular disorders: insights from bidirectional and multivariable Mendelian randomization analysis

**DOI:** 10.3389/fmed.2024.1431170

**Published:** 2024-10-09

**Authors:** Shipei Fan, Xing-yu Shi, Xia Li, Jun Li, Song-ping Yu

**Affiliations:** ^1^Department of Ophthalmology, Lishui Municipal Central Hospital, The Fifth Affiliated Hospital of Wenzhou Medical University, Zhejiang, China; ^2^Department of Nephrology, Lishui Municipal Central Hospital, The Fifth Affiliated Hospital of Wenzhou Medical University, Zhejiang, China

**Keywords:** vitamin D, 25(OH)D, ocular disorders, Mendelian randomization, causal relationship

## Abstract

**Purpose:**

This study aimed to assess the causal relationships between vitamin D levels and ocular disorders.

**Methods:**

Independent genetic variables were obtained from genome-wide association studies (GWAS) and publicly available databases. The summary statistics for 25-hydroxyvitamin D (25(OH)D) were obtained from two large-scale GWAS studies, with sample sizes of 324,105 and 417,580 European individuals. The genetic variants of myopia, primary open angle glaucoma (POAG), anterior iridocyclitis, senile cataract, diabetic retinopathy (DR), retinal vein occlusion (RVO), wet age-related macular degeneration (WAMD) and optic neuritis were extracted from the latest release of FinnGen consortium, which contains genome data from Finnish participants. Subsequently, Mendelian randomization (MR) analyses were conducted to obtain effect estimates. Additionally, we performed multivariable MR analysis and mediation analysis to validate the results.

**Results:**

In the discovery dataset, genetically predicted vitamin D concentration was found to be causally associated with an increased risk of WAMD, (odd ratio (OR) = 1.35, 95% confidence interval (CI) = 1.09–1.67, *P*_IVW_ = 0.005). However, no causal effects of genetically predisposed vitamin D levels on the risk of most types of ocular disorders were observed. Reverse MR revealed no causal relationships between the ocular diseases and vitamin D concentrations. The MR analyses of the validation dataset yielded consistent results. Additionally, the causal effect of vitamin D levels on the risk of WAMD remained significant after adjusting for potential confounders in the multivariable MR analysis (OR = 1.86, 95% CI = 1.26–2.73, *P*_IVW_ = 0.002).

**Conclusion:**

Our MR analysis results provide robust evidence of a causal relationship between genetically predicted 25(OH)D levels and an increased risk of WAMD in European population. These findings offer important insights into the management and control of ocular disorders.

## Introduction

Vitamin D, a fat-soluble vitamin, plays a crucial role in various biological pathways, including immune regulation, cell growth, inhibition of apoptosis and anti-angiogenesis (1, 2). Additionally, vitamin D is involved in maintaining endothelial cell function and exhibits anti-inflammatory effects in multiple autoimmune diseases (3, 4). Sunlight exposure is recognized as the primary source of endogenous synthesis of vitamin D3 in individuals (5). Dietary intake and supplements also contribute to the formation of vitamin D3 and vitamin D2. Both vitamin D3 and vitamin D2 undergo hydroxylation in the liver, converting them into 25-hydroxyvitamin D (25(OH)D). The serum level of 25(OH)D, which reflects skin production, dietary intake and supplementation, is considered as a reliable biomarker of vitamin D status (6).

In recent years, the mechanisms and associated pathogenesis of vitamin D in ocular diseases have attracted increasing attention worldwide. Extensive studies have explored the relationships between vitamin D and ocular disorders, spanning from the anterior segment to fundus (7). Myopia, a refractive error with rapidly increasing prevalence over the world, has been the subject of investigations (8, 9). A cohort study conducted in Western Australia demonstrated significantly reduced vitamin D levels in myopic individuals compared to nonmyopic populations, after the adjusting for confounding factors (10). Similarly, evidence from a large-scale epidemiological study on 6-year-old children suggests that decreased serum concentrations of 25(OH)D is closely associated with higher axial length and an increased risk of myopia (11). However, it is worth noting that Li and colleagues found no association between 25(OH)D levels and myopia in Chinese children and adolescents (12).

Diabetic retinopathy (DR), the most common ocular complication of diabetes, can lead to irreversible blindness (13). Previous study had shown that serum levels of 25(OH)D were significantly elevated in individuals with DR compared to diabetes patients without ocular lesions (14). Decreased vitamin D concentrations could serve as a potential biomarker and predictive factor for DR. Furthermore, some scholars have reported a positive association between 25(OH)D levels and the severity of DR, while Alam and colleagues opposed this finding (15–17). Age-related macular degeneration (AMD), a chronic disease and growing public health burden in industrialized countries, has also been widely investigated (18). A large-scale multicenter study suggested no linear relationship between 25 (OH)D levels and early AMD or neovascular AMD (19). In addition, evidence from a randomized clinical trial manifested no effect of vitamin D3 supplementation on the incidence of AMD (20). Overall, the associations between 25(OH)D and risk of ocular disorders remain controversial, due to the inevitable confounding bias of clinical investigations.

Mendelian randomization (MR) is a statistical approach used to examine causal relationships between exposure factors and outcomes. Since genes are randomly distributed in the process of inheritance, the relationship between risk factor and outcome will not be influenced by confounding factors. Taken the randomly assigned genetic variables during pregnancy into consideration, the direction of causal association can also be evaluated. Compared to conventional clinical trials, MR analysis could provide robust and potent evidence for causal inference. Therefore, the objective of this study was to comprehensively determine the causal relationship between serum vitamin D levels and ocular diseases. The findings of this study are expected to yield fresh insights and clinical implications for prevention and treatment strategies.

## Methods

### Study design

We conducted a comprehensive bidirectional MR analysis to explore the causal associations between vitamin D levels and ocular disorders. The present MR analysis was performed in accordance to the principles proposed by the STROBE-MR statement (21). Ethical approval and informed consent were not requested. To ensure robust and reliable results, the MR analysis should be conducted according to three basic assumptions: (1) the genetic variants as instrument variables (IVs) are associated with exposures strongly; (2) the IVs are not related to any potential confounders; and (3) the IVs affect the outcome only via exposure factors rather than other pathways and phenotypes. To minimize population stratification bias, the databases of exposures and outcomes were both from European population.

### Data for vitamin D and ocular disorders

The summary data for 25(OH)D was derived form a recently published genome-wide association study (GWAS) dataset, including phenotype, genotype and clinical demographics (22). This comprehensive large-scale study contained a total of 324,105 European individuals with self-reported fair skin, from the United Kingdom Biobank (UKB). To further identify the robustness of causality, we utilized another summary statistics for Vitamin D levels. This dataset contains 417,580 participants and body mass index (BMI) is considered as a covariate (23). The GWAS data for ocular disorders were collected from FinnGen research project, which is a public-private partnership aiming to analyze genome and health data from 500,000 Finnish biobank participants to understand disease mechanisms and predispositions (24). To ensure the reliability and completeness of the GWAS statistics, we extracted genetic variants from the latest data for various ocular diseases, including myopia (4,106 cases and 394,028 controls), primary open angle glaucoma (POAG: 8,530 cases and 391,275 controls), anterior iridocyclitis (7,152 cases and 405,029 controls), senile cataract (65,235 cases and 341,546 controls), DR (10,413 cases and 308,633 controls), retinal vein occlusion (RVO: 775 cases and 308,633 controls), wet AMD (WAMD, 5,239 cases and 273,920 controls) and optic neuritis (1,295 cases and 409,190 controls). Detailed characteristics of the included GWAS databases are summarized in Table 1.

**Table 1:** Characteristics of GWAS databases.

Trait	Resource	Year	Sample size	Data download
**Exposure**				
Vitamin D (Discovery)	Wang *et al*	2023	324,105 participants	DOI: 10.1371/journal.pgen.1011033
Vitamin D (Validation)	Revez *et al*	2020	417,580 participants	DOI: 10.1038/s41467-020-15421-7
HDL cholesterol	UK Biobank	2020	403,943 participants	gwas.mrcieu.ac.uk/datasets/ieu-b-109/
LDL cholesterol	UK Biobank	2020	440,546 participants	gwas.mrcieu.ac.uk/datasets/ieu-b-110/
Triglycerides	UK Biobank	2020	441,016 participants	gwas.mrcieu.ac.uk/datasets/ieu-b-111/
Type 2 diabetes	Xue *et al*	2018	62,892 cases and 596,424 controls	DOI: 10.1038/s41467-018-04951-w
Hypertension	UK Biobank	2018	119,731 cases and 343,202 controls	gwas.mrcieu.ac.uk/datasets/ukb-b-14057/
CRP	Said *et al*	2022	1,002,898 participants	DOI: 10.1038/s41467-022-29650-5
Cigarettes per day	Liu *et al*	2019	337,334 participants	DOI: 10.1038/s41588-018-0307-5
Alcoholic drinks per week	Liu *et al*	2019	1,232,091 participants	DOI: 10.1038/s41588-018-0307-5
**Outcome**				
Myopia	Finngen	2023	4,106 cases and 394,028 controls	r10.finngen.fi/
POAG	Finngen	2023	8,530 cases and 391,275 controls	r10.finngen.fi/
Anterior iridocyclitis	Finngen	2023	7,152 cases and 405,029 controls	r10.finngen.fi/
Senile cataract	Finngen	2023	65,235 cases and 341,546 controls	r10.finngen.fi/
Diabetic retinopathy	Finngen	2023	10,413 cases and 308,633 controls	r9.finngen.fi/
Retinal vein occlusion	Finngen	2023	775 cases and 308,633 controls	r9.finngen.fi/
WAMD	Finngen	2023	5,239 cases and 273,920 controls	r10.finngen.fi/
Optic neuritis	Finngen	2023	1,295 cases and 409,190 controls	r10.finngen.fi/

GWAS, genome-wide association study; CRP, C-reactive protein; POAG, primary open angle glaucoma; WAMD, wet age-related degeneration.

For the analysis, single nucleotide polymorphisms (SNPs) with genome-wide significance (*p* value <5 × 10^−8^), low linkage disequilibrium (*r*^2^ < 0.001) and a window size of 10,000 kb were selected when considering vitamin D levels as exposure. To investigate potential inverse relationships, we conducted bidirectional MR analysis by selecting SNPs at the genome-wide threshold of *p* value <5 × 10^−6^ when considering ocular diseases as exposures. We also performed linkage disequilibrium analysis to ensure independence among SNPs (*r*^2^ < 0.01 and clumping distance of 10,000 kb). Palindromic SNPs and SNPs with a minor allele frequency (MAF) less than 0.01 were excluded. Additionally, we used the F-statistic to evaluate the strength of the instrumental variables and avoid weak instrument bias, calculated using the equation: F = Beta^2^/SE^2^ (25). SNPs with a *F*-value less than 10 were further eliminated.

### MR analysis and sensitivity analysis

**Figure 1:**
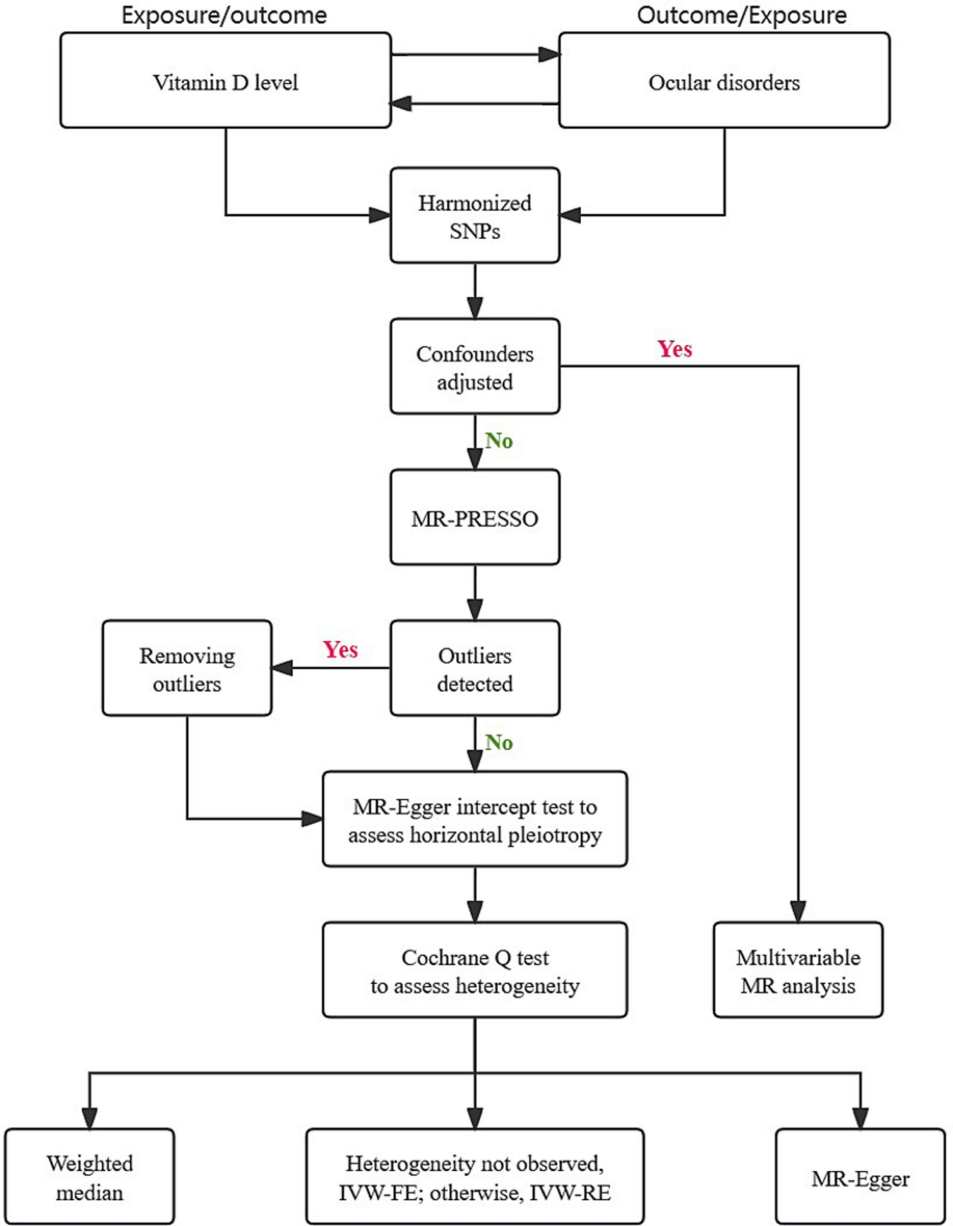
Flow chart of overall Mendelian randomization design.

The statistical analysis was conducted as depicted in Figure 1. After harmonizing the SNPs from exposures and outcomes, we employed the MR Pleiotropy RESidual Sum and Outlier (MR-PRESSO) test to identify and correct for potential pleiotropy by eliminating outliers (26). The Cochrane Q test was employed to assess heterogeneity between different genetic variants. The fixed-effects inverse-variance weighted (IVW) model was applied as the main MR analysis approach, providing robust and unbiased estimates (27). In case of significant heterogeneity, the alternative random-effects IVW method was conducted. The weighted median (WM) approach offers accurate effects even when up to 50% genetic variables are invalid (28). The MR-Egger regression was employed to provide an unbiased effect even when the majority of genetic instrument variables are invalid (29). In addition, we utilized the intercept of the MR-Egger test to examine horizontal pleiotropy.

Multivariable MR (MVMR) is a novel method incorporating genetic variations for potential confounders or mediators into the same model (30). If univariable MR analysis indicated a causal relationship, MVMR analysis was employed to adjust for confounders, including type 2 diabetes, hypertension, C-reactive protein (CRP), cigarettes per day and alcoholic drinks per week (31–33). Considering the lipid-soluble nature of vitamin D, we conducted mediation analysis using two-step MR to explore the potential mediating effects of high-density lipoprotein cholesterol (HDL-C), low-density lipoprotein cholesterol (LDL-C) and triglycerides on the causal relationships between vitamin D and ocular disorders. As presented in Table 1, the summary statistics of these phenotypes were obtained from the publicly available databases and large-scale GWAS studies.

All MR analyses were carried out by R-studio software (version 4.3.1), using the “TwoSampleMR,” “MendelianRandomization” and “MRPRESSO” packages. Considering multiple tests between exposures and outcomes, a Bonferroni-corrected threshold of *p* < 0.05/8 was considered statistically significant.

## Results

### Forward MR analysis

**Figure 2:**
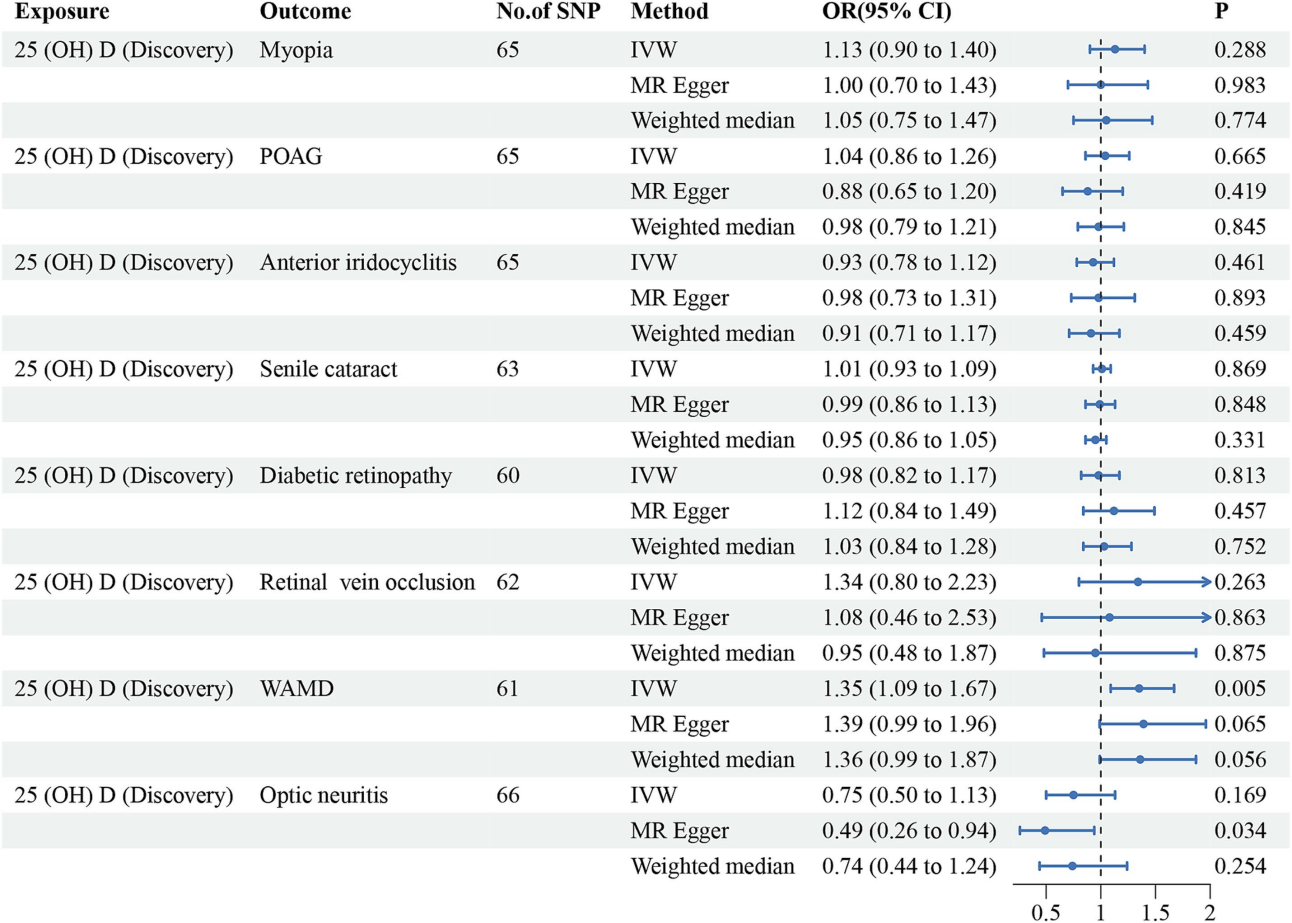
Forest plot for the causal effects of vitamin D (discovery dataset) on the risk of ocular disorders. SNP, single nucleotide polymorphism; 25(OH)D, 25-hydroxyvitamin D; POAG, primary open angle glaucoma; WAMD, wet age-related degeneration; IVW, inverse variance weighted; 95%CI, 95% confidence interval.

**Figure 3:**
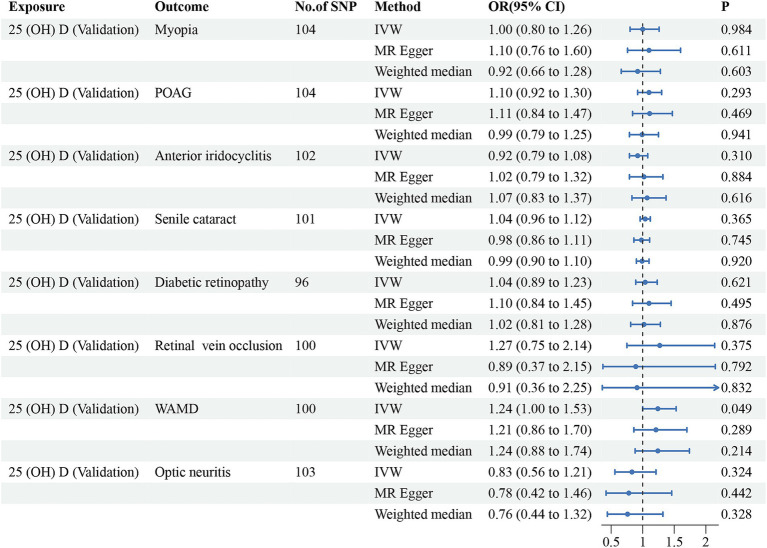
Forest plot for the causal estimates of vitamin D (validation dataset) on the risk of ocular disorders. SNP, single nucleotide polymorphism; 25(OH)D, 25-hydroxyvitamin D; POAG, primary open angle glaucoma; WAMD, wet age-related degeneration; IVW, inverse variance weighted; 95%CI, 95% confidence interval.

Initially, we conducted the MR-PRESSO test to identify potential pleiotropy and eliminate outliers of the discovery dataset. After eliminating the outliers, a total of 60–66 SNPs were finally included for further analysis. All of the included instrumental variants exhibited an F-statistic greater than 10, indicating their strong instrumental strength. The results, as summarized in Table 2 and Figure 2, revealed no significant associations between vitamin D levels and risk of myopia (odd ratio (OR) = 1.13, 95% confidence interval (CI) = 0.90–1.40, *P*_IVW_ = 0.288), POAG (OR = 1.04, 95% CI = 0.86–1.26, *P*_IVW_ = 0.665), anterior iridocyclitis (OR = 0.93, 95% CI = 0.78–1.12, *P*_IVW_ = 0.461), senile cataract (OR = 1.01, 95% CI = 0.93–1.09, *P*_IVW_ = 0.869), DR (OR = 0.98, 95% CI = 0.82–1.17, *P*_IVW_ = 0.813), RVO (OR = 1.34, 95% CI = 0.80–2.23, *P*_IVW_ = 0.263) and optic neuritis (OR = 0.75, 95% CI = 0.50–1.13, *P*_IVW_ = 0.169). However, a positive association was observed between genetically predicted vitamin D concentrations and an increased risk of WAMD (OR = 1.35, 95% CI = 1.09–1.67, *P*_IVW_ = 0.005). Similar trends were observed using WM and MR-Egger models, although statistical significance was not achieved. To validate these observations, we utilized another GWAS database for 25(OH)D. Outliers for anterior iridocyclitis (rs8107974), senile cataract (rs2229742) and DR (rs1260326) were identified and subsequently discarded. Employing the IVW method, a significant causal relationship was found between genetically assessed 25(OH)D levels and a higher risk of WAMD (OR = 1.24, 95% CI = 1.00–1.53, *P*_IVW_ = 0.049). However, this causal association did not retain statistical significance after Bonferroni correction, suggesting potential causality. Consistently with discovery analyses, no causal relationships were identified between the remaining ocular disorders and vitamin D levels. The comprehensive results of different models were presented in Table 2 and Figure 3. The MR-Egger regression intercept test indicated the absence of horizontal pleiotropy in the forward MR analysis (Table 2). The details of SNPs, including rs number, effect allele, other allele, effect allele frequency, beta value, standard error, *p*-value and F-statistics were presented in Supplementary Table S1.

**Table 2:** Mendelian randomization analyses between vitamin D levels and ocular disorders.

Exposure	Outcome	IVW		Heterogeneity		Pleiotropy	
		OR (95% CI)	*p* value	IVW Q	*p* value	Intercept	*p* value
25(OH)D (Discovery)	Myopia	1.13 (0.90, 1.40)	0.288	76.78	0.131	0.005	0.398
	POAG	1.04 (0.86, 1.26)	0.665	113.67	0.0001	0.007	0.173
	AI	0.93 (0.78, 1.12)	0.461	87.28	0.028	−0.002	0.687
	SC	1.01 (0.93, 1.09)	0.869	92.53	0.007	0.0009	0.711
	DR	0.98 (0.82, 1.17)	0.813	104.42	0.0002	−0.006	0.267
	RVO	1.34 (0.80, 2.23)	0.263	72.93	0.141	0.010	0.533
	WAMD	1.35 (1.09, 1.67)	0.005*	75.85	0.081	−0.001	0.844
	Optic neuritis	0.75 (0.50, 1.13)	0.169	83.72	0.059	0.019	0.103
25(OH)D (Validation)	Myopia	1.00 (0.80, 1.26)	0.984	138.46	0.011	−0.003	0.531
	POAG	1.10 (0.92, 1.30)	0.293	157.90	0.0004	−0.0004	0.911
	AI	0.92 (0.79, 1.08)	0.310	115.64	0.151	−0.004	0.335
	SC	1.04 (0.96, 1.12)	0.365	152.01	0.0006	0.002	0.267
	DR	1.04 (0.89, 1.23)	0.621	136.92	0.003	−0.002	0.627
	RVO	1.27 (0.75, 2.14)	0.375	119.33	0.080	0.012	0.331
	WAMD	1.24 (1.00, 1.53)	0.049*	134.99	0.009	0.001	0.845
	Optic neuritis	0.83 (0.56, 1.21)	0.324	125.29	0.059	0.002	0.828

IVW, inverse-variance weighted; OR, odds ratio; CI, confidence interval; 25(OH)D, 25-hydroxyvitamin D; POAG, primary open angle glaucoma; AI, anterior iridocyclitis; SC, senile cataract; DR, diabetic retinopathy; RVO, retinal vein occlusion; WAMD, wet age-related degeneration.

### Reverse MR analysis, MVMR analysis, and mediation analysis

Moving on to the reverse MR analysis, after harmonizing and MR-PRESSO test, the detailed characteristics of genetic variants were listed in Supplementary Table S2. The *F*-value of each genetic variable exceeded 10, indicating the absence of weak instrument bias. To ensure the robustness and reliability of MR results, at least 5 SNPs were utilized for each exposure. In relation to the discovery and validation datasets of vitamin D, various MR methods revealed no causal links between ocular disorders and levels of 25(OH)D (Supplementary Figures S1, S2). No evidence of horizontal pleiotropy was discovered through the MR-Egger intercept, thus confirming the reliability of results obtained from the reverse analysis (Supplementary Table S3).

Furthermore, we conducted MVMR analysis to assess the robustness of the significant causal relationship between vitamin D and WAMD. This analysis aimed to evaluate the potential effects of type 2 diabetes, hypertension, CRP, cigarettes per day and alcoholic drinks per week. The estimates from the discovery MVMR analysis revealed the causal association remained significant (Table 3, OR = 1.86, 95% CI = 1.26–2.73, *P*_IVW_ = 0.002). Consistent results were also observed using the WM and MR-Egger models (*p* < 0.05) and the MVMR-Egger intercept test demonstrated the absence of horizontal pleiotropy (Egger intercept = 0.003, *p* = 0.325). In the validation MVMR analysis, genetically determined 25(OH)D levels were found to be not associated with the risk of WAMD, after adjusting for potential confounders. Additionally, using the lipid parameters as mediators, no significant mediation relationship was found (Supplementary Table S4).

**Table 3:** Multivariable MR on the causal associations between 25(OH)D levels and WAMD.

Exposure	Outcome	Number of SNPs	Method	OR (95% CI)	*p* value
25(OH)D (Discovery)	WAMD	227	IVW	1.86 (1.26, 2.73)	0.002
		227	MR-Egger	1.85 (1.25, 2.73)	0.002
		227	Weighted median	1.87 (1.20, 2.92)	0.006
25(OH)D (Validation)	WAMD	238	IVW	1.11 (0.75, 1.65)	0.590
		238	MR-Egger	1.11 (0.75, 1.63)	0.614
		238	Weighted median	1.02 (0.63, 1.64)	0.943

25(OH)D, 25-hydroxyvitamin D; WAMD, wet age-related degeneration; OR, odds ratio; CI, confidence interval; IVW, inverse-variance weighted.

## Discussion

To the best of our knowledge, no prior studies have comprehensively explored the causal associations between vitamin D and 8 types of ocular disorders. Our fundings indicate that genetically determined 25(OH)D levels are associated with an increased risk of WAMD, which is further validated by MVMR analysis. Additionally, reverse MR analysis demonstrated no causal relationships between ocular diseases and 25(OH)D concentrations.

The most noteworthy finding emerging from this MR analysis is the causal effect of vitamin D on an increased risk of WAMD, which should be interpreted with caution. The mechanisms of AMD remain not fully understood, but there is a consensus that dysfunction of retinal pigment epithelium (RPE) plays an essential role. RPE is characterized as a pivotal component of blood retinal outer barrier, participating in the phagocytosis of photoreceptor outer segments and scavenging the damaged ROS (34). Multiple risk factors have been identified in the development of AMD, including age, smoking, genetic factors and sunlight exposure (35). Geographic position and insolation have been identified as essential factors in the prevalence of AMD, as demonstrated by meta-regression analysis (36). It has been shown that ultraviolet and blue light can lead to the damage of RPE cells (37–39). A case–control study found that sunlight exposure during working life could increase the risk of early and late AMD (40). Individuals born in summer were observed to have a higher season-specific risk of neovascular AMD compared to those born in winter (41). Additionally, a study from the United States found a peak in 25(OH)D concentrations in August and a trough in February (42). Since sunlight exposure is the main source of vitamin D, we infer that additional sunlight exposure may increase the risk of WAMD. As described in previous MR analyses, the serum lipid biomarkers, CRP levels, smoking and alcohol intake are causally associated with AMD (43–47). To account for the potential effects of these confounders, we performed the MVMR and mediation analyses. The results indicated that the causal relationship remained significant after adjusting for confounders and no mediation effects of lipid parameters were observed, further validating the robustness of causal estimates.

As widely acknowledged, cross-sectional studies cannot determine whether deficiency of vitamin D is the cause or result of diseases. In view of several unavoidable bias in clinical trials, the evidence from previous investigations for the relationships between vitamin D and risk of ocular diseases is controversial. From the perspective of genetics, we confirmed there were no causal links between vitamin D and other types of ocular disorders. A published MR analysis indicates vitamin D levels contribute little to the degree of myopia, while time spent outdoors is identified as an essential confounder (48). As previous reported, dopamine plays critical roles in the retina development and visual signaling (49). It has been suggested that time spent outdoors, resulting in sunlight exposure, prevents myopia through the dopamine-mediated pathway (50). Glaucoma, a leading cause of irreversible visual impairment, is characterized by the damage to optic nerve head and visual field (51). As previously reported, a decreased 25(OH)D concentration was associated with a higher risk of POAG (52, 53). The investigators speculate that the status of vitamin D deficiency may deteriorate the biological functions of optic nerve through oxidative stress pathway. A meta-analysis has manifested no significant difference in serum vitamin D levels between glaucoma patients and healthy controls, which is consistent with our genetic results (54). Further work is required to investigate the detailed mechanisms of vitamin D in various subtypes of glaucoma.

Prior meta-analyses have revealed positive correlations between vitamin D deficiency and a higher risk of non-infectious uveitis (55). Vitamin D has been demonstrated to paly vital roles in immune responses and gut microbiota composition (56). The deficiency of vitamin D may lead to immune dysregulation and imbalance of gut microbiota, potentially activating and accelerating the progression of uveitis. However, the majority of included studies in meta-analysis are designed as case–control and cross-sectional, limiting the robustness of results. Additionally, a two-sample MR analysis indicates positive causal effects of low vitamin D levels on the risk of uveitis (57). The GWAS information used in the present MR analysis was extracted from anterior iridocyclitis patients, so the discrepancy of MR results could be attribute to variations in characteristics among different GWAS databases. To elucidate the pathogenesis of vitamin D in cataract, a random controlled trial was performed in 23,315 older Australian adults (58). The authors found that high-dose routine vitamin D supplementation had no effect on the need for cataract surgery, which is in accordance with our MR results.

Retrospective studies reported no significant relationship between vitamin D status and DR after controlling for confounding factors (17, 59). The authors also found that majority of DR patients were lack of vitamin D, the association between degree of retinopathy and vitamin D could not be fully investigated. Regarding the RVO and optic neuritis, there remains a paucity of valid evidence. A case–control study showed no differences in vitamin D levels between central RVO patients and healthy controls (60). In addition, data from a randomized and placebo-controlled trial demonstrated that oral supplementation of vitamin D had no significant effect on the thickness of retinal nerve fiber layer in optic neuritis patients (61). It is worthwhile to point out that the sample sizes of these studies are relatively small, longitudinal and prospective trials are warranted to elucidate our results in the future.

Some strengthens of this MR analysis are worth noting. First, the MR design ensures the strengthen of genetic variables, reducing the influence of potential confounders in observational studies and providing a robust estimate of causal associations. Second, the relatively large sample size in the MR analysis allows for reliable and accurate causal relationships. However, several certain limitations should be considered. First, the genetic instruments extracted from GWAS data are from European individuals, the results of this study may not be explainable in other populations and races. Additionally, despite employing various approaches to evaluate and adjust for possible confounding and pleiotropic effects, potential confounding factors could not be completely ruled out. Taken the complex biological pathways of Vitamin D into consideration, the results of MR analysis were acquired via the genetic pathway, which may only represent a specific perspective. Moreover, due to the limited demographics and characteristics of the GWAS data, performing subgroup analyses seemed challenging, limiting the comprehensiveness of the present MR analysis.

In conclusion, genetically predicted vitamin D was causally associated with an enhanced risk of WAMD in the European population. Additionally, based on the current MR analysis, we found no convincing evidence of causal links between vitamin D concentrations and most types of ocular disorders. Our results provide novel insights into the prevention and management of ocular diseases. Further randomized controlled trials are needed to evaluate and confirm our findings in the future.

## Data Availability

The original contributions presented in the study are included in the article/Supplementary material, further inquiries can be directed to the corresponding authors.
