# Supplementary figures and images for: Vitamin D levels and risk of ocular disorders: insights from bidirectional and multivariable Mendelian randomization analysis
Source: Front Med (Lausanne). 2024 Oct 9;11:1431170. doi: 10.3389/fmed.2024.1431170 (PMC11496056; doi:10.3389/fmed.2024.1431170)

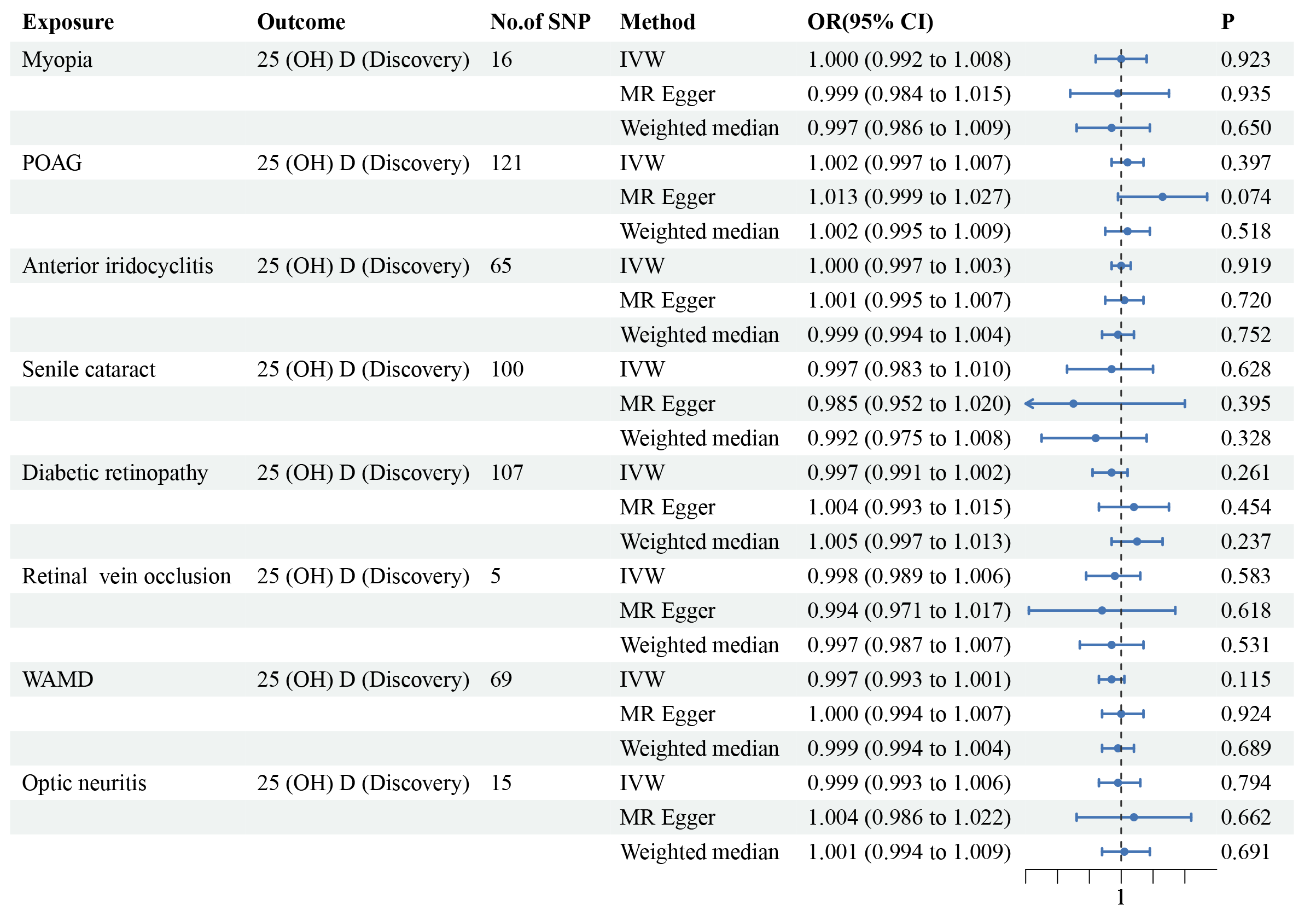

Supplement: Supplementary file 1 [file Image_1.TIF]

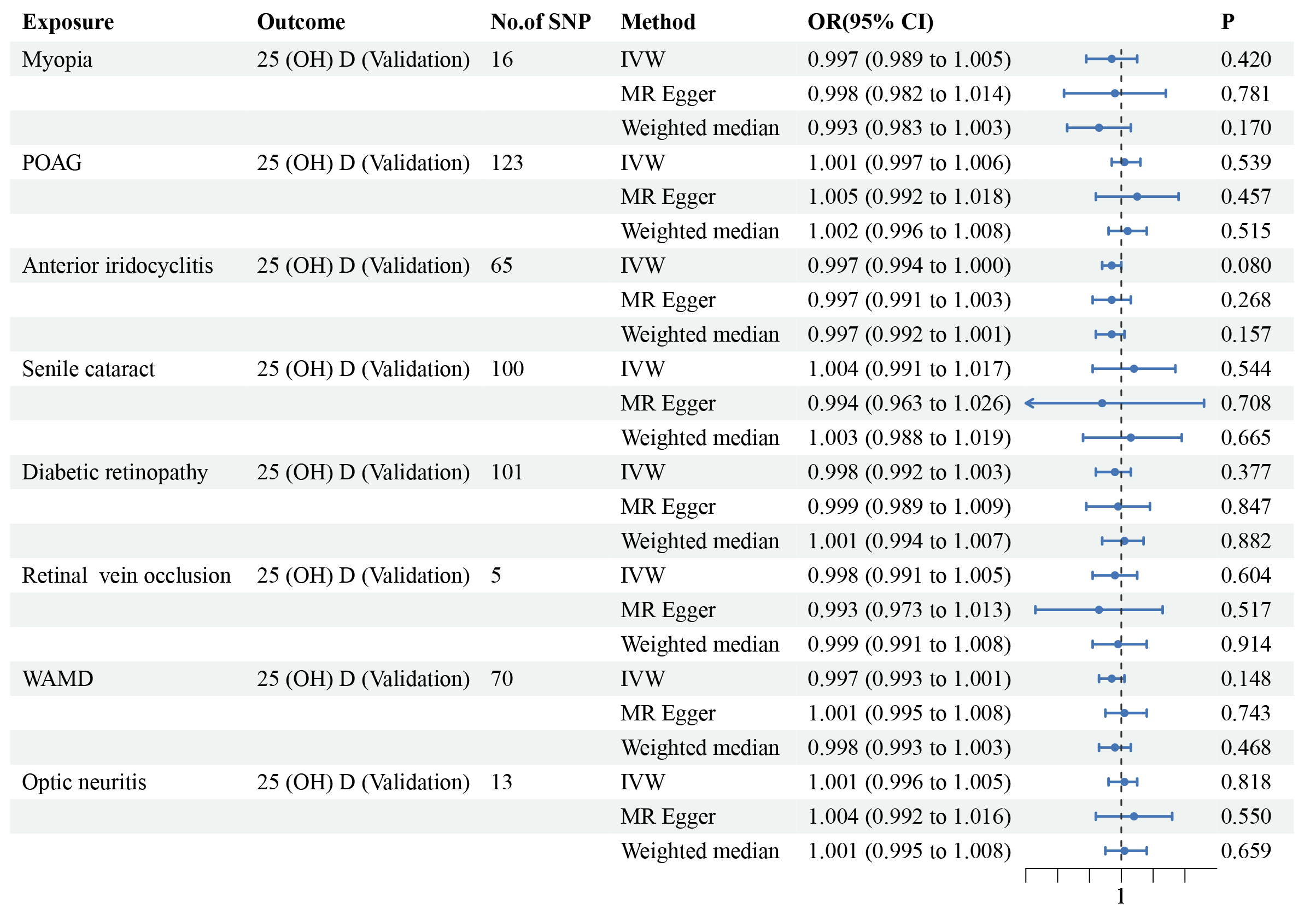

Supplement: Supplementary file 2 [file Image_2.TIF]
