# Supplementary material for: Vitamin D levels and risk of ocular disorders: insights from bidirectional and multivariable Mendelian randomization analysis
Source: Front Med (Lausanne). 2024 Oct 9;11:1431170. doi: 10.3389/fmed.2024.1431170 (PMC11496056; doi:10.3389/fmed.2024.1431170)
Supplement: Supplementary file 5 [file Table_3.docx]

**Table S3. Mendelian randomization analyses between ocular disorders and vitamin D levels**

| Exposure | Outcome | IVW | | Heterogeneity | | Pleiotropy | | |
| --- | --- | --- | --- | --- | --- | --- | --- | --- |
|  |  | OR (95% CI) | *P* value | IVW Q | *P* value | | Intercept | *P* value |
| Myopia | 25 (OH) D (Discovery) | 1.000 (0.992, 1.008) | 0.923 | 14.53 | 0.485 | | -0.0002 | 0.715 |
| POAG |  | 1.002 (0.997, 1.007) | 0.397 | 146.95 | 0.048 | | -0.001 | 0.111 |
| AI |  | 1.000 (0.997, 1.003) | 0.919 | 64.32 | 0.465 | | 0.0003 | 0.878 |
| SC |  | 0.997 (0.983, 1.010) | 0.628 | 180.08 | 1.18 x10^-6^ | | 0.0007 | 0.473 |
| DR |  | 0.997 (0.991, 1.002) | 0.261 | 184.22 | 3.83 x10^-6^ | | -0.001 | 0.119 |
| RVO |  | 0.998 (0.989, 1.006) | 0.583 | 1.16 | 0.884 | | 0.002 | 0.729 |
| WAMD |  | 0.997 (0.993, 1.001) | 0.115 | 84.34 | 0.087 | | -0.0009 | 0.230 |
| Optic neuritis |  | 0.999 (0.993, 1.006) | 0.794 | 23.10 | 0.059 | | -0.002 | 0.572 |
|  |  |  |  |  |  | |  |  |
| Myopia | 25 (OH) D (Validation) | 0.997 (0.989, 1.005) | 0.420 | 20.55 | 0.152 | | -0.0003 | 0.882 |
| POAG |  | 1.001 (0.997, 1.006) | 0.539 | 190.16 | 7.71 x10^-5^ | | -0.0004 | 0.676 |
| AI |  | 0.997 (0.994, 1.000) | 0.080 | 81.00 | 0.074 | | 0.0001 | 0.863 |
| SC |  | 1.004 (0.991, 1.017) | 0.544 | 202.07 | 4.75 x10^-9^ | | 0.0006 | 0.502 |
| DR |  | 0.998 (0.992, 1.003) | 0.377 | 193.91 | 5.60 x10^-8^ | | -0.0002 | 0.756 |
| RVO |  | 0.998 (0.991, 1.005) | 0.604 | 0.57 | 0.967 | | 0.002 | 0.601 |
| WAMD |  | 0.997 (0.993, 1.001) | 0.148 | 108.17 | 0.002 | | -0.001 | 0.164 |
| Optic neuritis |  | 1.001 (0.996, 1.005) | 0.818 | 9.55 | 0.655 | | -0.001 | 0.579 |

IVW: inverse-variance weighted; OR: odds ratio; CI: confidence interval; 25 (OH) D: 25-hydroxyvitamin D; POAG: primary open angle glaucoma; AI: anterior iridocyclitis; SC: senile cataract; DR: diabetic retinopathy; RVO: retinal vein occlusion; WAMD: wet age-related degeneration.
