# Supplementary material for: Vitamin D levels and risk of ocular disorders: insights from bidirectional and multivariable Mendelian randomization analysis
Source: Front Med (Lausanne). 2024 Oct 9;11:1431170. doi: 10.3389/fmed.2024.1431170 (PMC11496056; doi:10.3389/fmed.2024.1431170)
Supplement: Supplementary file 6 [file Table_4.DOCX]

**Table S4. Mediation analyses using the two-step MR**

| Exposure | Mediator | Outcome | Number of SNPs | Odds ratio | P value |
| --- | --- | --- | --- | --- | --- |
| 25 (OH) D (Discovery) | HDL-C |  | 74 | 1.018 | 0.305 |
|  | HDL-C | WAMD | 325 | 1.239 | 0.001 |
| 25 (OH) D (Discovery) | LDL-C |  | 72 | 0.999 | 0.949 |
|  | LDL-C | WAMD | 163 | 1.081 | 0.258 |
| 25 (OH) D (Discovery) | TG |  | 69 | 0.938 | 0.002 |
|  | TG | WAMD | 281 | 0.826 | 0.001 |
|  |  |  |  |  |  |
| 25 (OH) D (Validation) | HDL-C |  | 43 | 1.010 | 0.525 |
|  | HDL-C | WAMD | 326 | 1.241 | 0.001 |
| 25 (OH) D (Validation) | LDL-C |  | 46 | 0.997 | 0.871 |
|  | LDL-C | WAMD | 163 | 1.081 | 0.258 |
| 25 (OH) D (Validation) | TG |  | 45 | 0.957 | 0.042 |
|  | TG | WAMD | 281 | 0.826 | 0.001 |

25 (OH) D: 25-hydroxyvitamin D; HDL-C: high-density lipoprotein cholesterol; LDL-C: low-density lipoprotein cholesterol; TG: triglycerides; WAMD: wet age-related degeneration.
